# Barriers and Facilitators to Surgical Trainee Psychological Safety

**DOI:** 10.1001/jamanetworkopen.2025.34462

**Published:** 2025-09-29

**Authors:** Jennifer H. Chen, Alyssa A. Pradarelli, Julie Evans, Niki Matusko, Norah N. Naughton, Roy Phitayakorn, John T. Mullen, Lily Chang, Melissa Johnson, Thavam Thambi-Pillai, Jon Ryckman, Melissa Alvarez-Downing, Nell Maloney Patel, Sebastiano Cassaro, Felicia Ivascu, David T. Hughes, Gurjit Sandhu

**Affiliations:** 1 Department of Surgery, Baylor College of Medicine, Houston, Texas; 2 Department of Surgery, Center for Surgical Training and Research, University of Michigan, Ann Arbor; 3 Office of Well-Being, University of Michigan, Ann Arbor; 4 Department of Anesthesiology, University of Michigan, Ann Arbor; 5 Department of Surgery, Massachusetts General Hospital, Boston; 6 Department of Surgery, Virginia Mason Medical Center, Seattle, Washington; 7 Department of Surgery, Gundersen Health System, La Crosse, Wisconsin; 8 Department of Surgery, University of South Dakota Sanford School of Medicine, Sioux Falls; 9 Department of Surgery, Rutgers New Jersey Medical School, Newark; 10 Department of Surgery, Rutgers Robert Wood Johnson Medical School, New Brunswick, New Jersey; 11 Department of Surgery, Kaweah Health, Visalia, California; 12 Department of Surgery, Oakland University William Beaumont School of Medicine, Royal Oak, Michigan; 13 Department of Surgery, University of Michigan, Ann Arbor

## Abstract

**Importance:**

Although psychological safety has been shown to be imperative for effective learning, specific factors associated with its presence in surgical training have not been well characterized.

**Objective:**

To identify barriers and facilitators to psychological safety for surgical trainees in the clinical learning environment.

**Design, Setting, and Participants:**

This qualitative study was conducted from February 20 to May 2, 2024, among 25 trainees in general surgery and 3 surgical subspecialty programs at 9 teaching hospitals—4 university-based, 3 community-based, and 2 community-based, university-affiliated hospitals.

**Exposure:**

Participation in surgical training.

**Main Outcomes and Measures:**

Participants were asked to share their perspectives and experiences in training in association with psychological safety in the learning environment. Purposive sampling was used to recruit for semistructured interviews. Transcripts were coded inductively using an interpretive description method.

**Results:**

Of 25 surgical trainees (13 women [52%]), 13 (52%) were junior residents (postgraduate years 1-3) and 7 (28%) were members of racial and ethnic minority groups. Four barriers and 3 facilitators to psychological safety were identified. Trainees cited (1) public humiliation, (2) tension around questioning, (3) culture fostered by faculty, and (4) influence of flawed system factors as barriers to psychological safety. Learners described instances of mistreatment and public shaming as detrimental to establishing psychological safety. Although trainees expressed a desire to ask more questions, fear of judgment served as a major barrier to building psychological safety through question asking in the trainee-faculty relationship. Although faculty played a key role in dictating whether psychological safety was present, trainees also noted overarching system-level factors, such as understaffing and lack of resources, as obstacles to promoting psychological safety. Conversely, (1) the work involved in promoting psychological safety, (2) trust, and (3) normalizing vulnerability in the clinical learning environment were facilitators of psychological safety. Trainees highlighted intentionality of actions by chief residents and faculty that explicitly promoted psychological safety. Instances where trust was initially “on the line” (ie, at serious risk of being lost) but ultimately reinforced formed the basis for psychological safety. Furthermore, acknowledgment of shortcomings normalized open sharing of mistakes in a psychologically safe manner.

**Conclusions and Relevance:**

This qualitative study identified 7 key individual, interpersonal, and organizational factors associated with psychological safety for surgical trainees. These findings may help inform the future development of interventions aimed at promoting psychological safety in the learning environment for surgical trainees.

## Key Points

**Question:**

What are potential barriers and facilitators to psychological safety for surgical trainees in the clinical learning environment?

**Findings:**

In this qualitative study of 25 surgical trainees across 9 residency programs, 4 barriers and 3 facilitators to psychological safety were identified. Barriers consisted of (1) public humiliation, (2) tension around questioning, (3) culture fostered by faculty, and (4) influence of flawed system factors; facilitators included (1) the work involved in promoting psychological safety, (2) trust, and (3) normalizing vulnerability in the clinical learning environment.

**Meaning:**

Findings highlight 7 key individual, interpersonal, and system-level factors associated with psychological safety for surgical trainees, which may inform the development of future interventions.

## Teaser

This qualitative study characterizes barriers and facilitators to psychological safety for surgical trainees in the clinical learning environment.

## Introduction

In recent years, the clinical learning environment in surgical training has faced increasing scrutiny due to greater emphasis on trainee well-being.^1,2,3^ The presence of psychological safety, where individuals feel comfortable speaking up without fear of repercussions, has been proposed as a prerequisite for effective learning and trainee inclusivity.^4,5,6,7,8^ Unfortunately, in the high-stakes, high-stress, hierarchical nature of surgical training, fostering an environment of psychological safety can be difficult. Specifically, fear of failure, constant performance assessments, and power differentials are inherent to medical training and may hinder the development of a psychologically safe educational space.^8,9,10^

High levels of psychological safety have been shown to increase work engagement, team performance, and job satisfaction within industrial and health care organizations.^7,11,12^ However, psychological safety and components of its makeup remain poorly defined in medical education. Limited studies have linked higher levels of psychological safety with increased resident satisfaction and professional fulfillment, greater adverse event reporting, higher perceived program support, and decreased rates of burnout.^13,14,15,16,17,18^ Despite these findings, there exists a knowledge gap on ways to optimize psychological safety within surgical training. Better understanding of psychological safety and identification of key barriers and facilitators may positively affect the growth and development of surgical trainees, as well as team dynamics, and ultimately improve patient outcomes.

To better understand the current state of psychological safety in surgical education, it is imperative to first understand what enables as well as hinders its promotion.^18^ The goal of this study is to qualitatively characterize barriers and facilitators of psychological safety for surgical trainees in the clinical learning environment.

## Methods

### Study Design

This was a qualitative study embedded within a 4-phase mixed-methods study design: (1) quantitative survey, (2) qualitative interviews, (3) participatory action research, and (4) program evaluation. The overarching goal of the parent study aims to characterize the associations of psychological safety with the learning environment and well-being among surgical trainees and faculty through explanatory sequential design. The current study reported here represents an exploratory analysis of the qualitative phase to further explore barriers and facilitators of psychological safety for surgical trainees. Semistructured interviews were conducted from February 20 to May 2, 2024, across 9 general surgery residency programs. Verbal informed consent was obtained from all participants prior to the start of the interview. All interview participants were offered a $25 gift card as compensation. This study was approved by the University of Michigan institutional review board, and results follow the Consolidated Criteria for Reporting Qualitative Research (COREQ) reporting guideline.^19^

### Participant Recruitment

Purposive sampling was used for recruitment. Recruitment emails were distributed with the assistance of program directors and administrative staff at the respective programs. Two reminder emails were sent 1 week apart. Sample size was guided by principles of information power, which appraises the adequacy of sample size based on the characteristics and quality of information a potential study population holds, rather than a numerical value.^20^ Sufficient information power from the study sample was determined based on 5 key elements: (1) study aim, (2) sample specificity, (3) use of established theory, (4) quality of dialogue, and (5) analysis strategy. Given the study’s narrow aim, the sampling pool’s high specificity, the presence of a theoretical background from our prior quantitative study, adequate dialogue quality, and the exploratory nature of the analysis strategy, the final study cohort was deemed to contain sufficient information power.

### Data Collection

The semistructured interview guide was developed through an iterative review process and pilot testing. Questions were developed based on previously identified learning environment constructs that were positively and negatively correlated with psychological safety.^18^ A pilot study was conducted at a single institution with 2 trainees to gather participant feedback. The purpose of the pilot study was to assess the feasibility and acceptability of the interview guide and the overall study approach. Two surgical trainees participated in one-on-one 45-minute interviews followed by 15-minute feedback sessions. The content and presentation of the interview guide were deemed appropriate and easily comprehensible by the pilot phase participants. The final 11-item interview guide is included in the eAppendix in Supplement 1. One-on-one 30- to 45-minute interviews were conducted virtually by 3 study team members (J.H.C., A.A.P., and J.E.). Interview transcripts were deidentified and reviewed against audio recordings to ensure accuracy. A demographic survey was administered via Qualtrics after interviews to collect participant self-reported gender, race and ethnicity (Asian, non-Hispanic Black, and non-Hispanic White), institution, surgical specialty, and clinical postgraduate year. Race and ethnicity data were collected to allow examination of potential variation in perceived barriers and facilitators of psychological safety as experienced by different demographic groups. Residents on academic development time were asked to answer questions based on prior clinical experiences.

### Data Analysis

An interpretive description method was used for thematic analysis.^21^ Interviews were coded inductively in NVivo, version 14 (Lumivero). Three authors (J.H.C., A.A.P., and J.E.) independently coded the first 3 transcripts and met after completion of each transcript to compare and ensure consistency in coding approach. The remaining transcripts were coded independently by 2 authors (J.H.C. and A.A.P.). A preliminary codebook was developed and iteratively revised after the completion of the coding of every 5 transcripts. Study members (J.H.C., A.A.P., and G.S.) met at regular intervals to review coding, resolve discrepancies, and identify themes. Two authors (J.H.C. and A.A.P.) are current or prior general surgery residents, and 2 authors (J.E. and G.S.) are qualitative researchers. Reflexivity was practiced throughout the coding process to reduce bias in data interpretation and reporting of themes. Investigator triangulation—achieved through involvement of multiple team members in data collection (J.H.C., A.A.P., and J.E.), independent analysis of interview data by multiple coders, and collaborative discussion for consistency in the analysis—enhanced the credibility and rigor of our findings by minimizing individual bias and promoting a more comprehensive interpretation of the data.

## Results

### Participant Demographics

In total, 25 trainees (13 women [52%] and 12 men [48%]) were interviewed (Table 1). Of the participants, 13 (52%) were junior residents (postgraduate years 1-3), and 4 (16%) were Asian, 3 (12%) were non-Hispanic Black, and 18 (72%) were non-Hispanic White. Most trainees (22 [88%]) were in general surgery residency programs, while the remaining (3 [12%]) were in subspecialty programs. More than half of the trainees (13 [52%]) were from university-based programs, while 8 (32%) were from community-based programs and 4 (16%) were from community-based, university-affiliated programs.

**Table 1.** Participant Characteristics

Characteristic	No. (%) (N = 25)
Gender	
Female	13 (52)
Male	12 (48)
Race and ethnicity	
Asian	4 (16)
Non-Hispanic Black	3 (12)
Non-Hispanic White	18 (72)
Clinical PGY level	
1	8 (32)
2	2 (8)
3	3 (12)
4	2 (8)
5	5 (20)
Research	4 (16)
Fellow	1 (4)
Surgical specialty	
General surgery	22 (88)
Other subspecialty	3 (12)
No. of institutions	
Community based	3
Community based, university affiliated	2
University based	4
Participants per institution	
Community based	8 (32)
Community based, university affiliated	4 (16)
University based	13 (52)

Abbreviation: PGY, postgraduate year.

### Barriers to Psychological Safety

Four barriers to psychological safety were identified: (1) public humiliation, (2) tension around questioning, (3) culture fostered by faculty, and (4) influence of flawed system factors (Table 2).

**Table 2.** Thematic Analysis Summary: Barriers to Psychological Safety

Themes	Definition	Representative quotes
Public humiliation	Moments of public shaming after speaking up or in response to mistakes led to feelings of being a failure	“His MO was ridiculing you in the OR; if you don’t know how to do something, and if you make mistakes, then he just takes it away from you instead of trying to correct you. They don’t have a clear interest in your education… once you do make a mistake, instead of doing what other attendings do where they try to educate you, they just ridicule you…” (Participant 104) “For the next hour, I stood in the room, watched him operate, while every few minutes [he] threw pretty demeaning comments about how it is completely inappropriate to train at their hospital and have that privilege without knowing all the details of their patients, and daring to come scrub with them.” (Participant 109) “You could just imagine those repeated insults over time, because you’re never going to feel good. It’s never, it’s never going to build you up to be a good person and a better version of yourself. If anything, that is going to turn you into someone who’s callous and propagate forward this negative cycle…” (Participant 115)
Tension around questioning	Trainees described reluctance to ask questions due to fear of judgment from faculty and negative experiences of being looked down upon when questions were answered incorrectly	Fear of judgment: “I think sometimes stress, intraoperatively manifests in different ways, but I feel like the couple of attendings that I can think of that I don’t enjoy operating with are people who yell or raise their voice, and it doesn’t… the whole room is so unsettling from the minute the incision is made, until we’re closed that it doesn’t feel appropriate to ask a question, even if I am unsure about something because I don’t know… because I feel like it’s not going to be well perceived.” (Participant 106) Lack of questions: “The amount of question asking to residents has probably gone down quite a bit as well. But I think there’s a bit of missing out on those learning opportunities at the expense of inclusivity and feeling comfortable, because I could feel comfortable standing by myself in a case silently throughout the whole thing watching. I mean, it’s comfortable… I’m not being engaged, and I won’t remember it.” (Participant 101) Negative response in questioning: “I don’t want to not be asked questions. I just don’t want to be made to feel like a failure because I got one wrong. I’m here to learn; therefore, I must not know everything. So, we cannot expect me to get all your questions right. If that’s the expectation, we are starting off on the wrong foot.” (Participant 113)
Culture fostered by faculty	Negative culture fostered by faculty and lack of prioritizing psychological safety trickles down to junior residents and sets the tone for the entire team	Detrimental behaviors and practices: “I think […] that the attending has a reputation of not listening to people [or] sometimes can lose their temper in the operating room. I think going into the case you’re kind of on edge and you’re prepared for that confrontation. So, you’re just waiting for it to happen, inevitably whenever it happens during the case.” (Participant 107) Psychological safety not prioritized: “Some people when they go to the OR, they enter a zone. Some attendings when they operate, they’re different than when they’re outside. In the OR, they’re a performance-oriented machine that needs to get things done. After the OR, they’re a far more pleasant person than in the OR because patient safety is important, things need to be done efficiently, and they know that they are the ones in control. And if something goes wrong, someone may actually get harmed. So, it’s a different operative metric. So, I think in the OR, patient safety is far more important than anything else.” (Participant 108) Trickle-down effect: “Something I’ve noticed is like the way that I see the attendings treat the chief makes me feel either like comfortable or uncomfortable. And so, I take a lot of cues from that. If they treat the chief in a very helpful and teaching manner, then I feel very comfortable asking a question that I don’t understand. Versus if they’re very punitive, then I’m not saying anything. I’m just hoping they don’t ask me to do anything because I don’t want to mess it up and then get in trouble.” (Participant 110)
Flawed system factors	System factors such as OR staff, support staff, psychosocial resources, and leadership changes that are detrimental to psychological safety	“There’s this unspoken judgment that I’m feeling from the scrub nurse that I’m incompetent or from the circulator who’s refusing to answer my pages. And I’m getting stressed because I don’t know what the page is about. And it might be about the patient who needs something and then like, do I need to scrub out? But then I’m the only person in this case with the attending, it’s like that sort of situation where that all culminates together, and I think it can feel pretty stressful, and then I close up and I feel like I can’t, I’m not at all, I don’t, I don’t feel comfortable, like speaking out, or learning.” (Participant 114) “I think that the isolation that came with COVID-19 was one of the most damaging components, as someone who was an intern during COVID-19, building relationships, and even feeling the faculty know your name. Now, this also translates into how they treat you, as well as other residents.” (Participant 102) “I think just like staffing, especially for on the floor and psychological safety and whatnot, when it comes to interactions with nurses and individuals, I think, you know, everyone’s feeling the pressures of not being able to have fully staffed parts and I think that sometimes comes back on the residents a little bit and it can definitely make you not want to speak up.” (Participant 115)

Abbreviations: MO, modus operandi; OR, operating room.

#### Public Humiliation

Trainees cited moments of public shaming and ridiculing by faculty as major detriments to psychological safety. Instances of public humiliation often occurred in response to trainees speaking up, clinical errors and misjudgment, or incorrect answers to questions. During these instances, trainees remembered the “lessons learned” due to the emotions associated with such interactions. However, the public nature of these encounters resulted in long-term loss of psychological safety and “propagated forward [a] negative cycle” of a strained trainee-faculty relationship.

#### Tension Around Questioning

Tensions related to questioning served as a major barrier to psychological safety through 3 subthemes: (1) fear of judgment, (2) lack of questions, and (3) negative response in questioning. Trainees highlighted the bidirectional nature of questioning, which involved asking questions of faculty and being asked questions by faculty. Trainees expressed constant fear of being judged for asking “bad” or “wrong” questions. There was an overall reluctance to ask questions due to fear of revealing knowledge deficits and being perceived negatively by faculty. Despite these fears, trainees also acknowledged a general lack of question asking by faculty. Although these interactions were more comfortable for trainees, they were ultimately detrimental to learning. Trainees emphasized that lack of question asking removed any risk taking between faculty and trainees. As 1 trainee stated, “I don’t feel like I’m asked nowhere near as many questions as I was elsewhere. And so, because of that, I don’t have a ton of experiences where I was asked a question, I got it wrong, and I was okay. So, then I don’t feel as safe asking my own questions, because I don’t know if I’m going to be judged for asking that question because it’s a dumb question.” Last, when trainees did respond to questions posed by faculty, negative reactions to wrong answers resulted in self-doubt, feelings of imposter syndrome, and future hesitancy to speak up.

#### Culture Fostered by Faculty

As the primary leaders within surgical teams, faculty played a major role in dictating the presence or absence of psychological safety through their beliefs, attitudes, and behaviors. Specifically, 3 subthemes related to culture fostered by faculty served as barriers: (1) detrimental behaviors and practices, (2) psychological safety not prioritized, and (3) trickle-down effect. Trainees cited behaviors such as impulsivity, impatience, lack of receptivity to questions, and overall lack of interest in teaching as barriers to psychological safety. However, trainees also noted instances in the operating room where “patient safety [was] far more important” than psychological safety, particularly within the high-stress operating room environment. In instances when faculty were perceived to be contributing to an unsupportive team culture, this sentiment was pervasive at all levels and trickled down to residents. Trainees also described taking cues from interactions between the chief resident and faculty, whereby the presence or absence of psychological safety dictated their own willingness to speak up.

#### Flawed System Factors

Trainees cited several system-level factors that served as barriers to psychological safety. On an interprofessional level, power dynamics between operating room staff and trainees served as a constant stressor. As 1 trainee stated, “There’s this unspoken judgment that I’m feeling from the scrub nurse that I’m incompetent or from the circulator who’s refusing to answer my pages.” Understaffing of nursing teams and lack of backup coverage among residents contributed to an already resource-limited and high-stress team setting, placing greater strain on team dynamics and on a willingness to challenge the status quo. More broadly, trainees noted a lack of resources to help resolve interpersonal conflicts. Last, social distancing during the COVID-19 pandemic also served as a major setback in establishing psychological safety.

### Facilitators of Psychological Safety

Three major facilitators of psychological safety were identified: (1) the work involved in promoting psychological safety, (2) trust, and (3) normalizing vulnerability in the clinical learning environment (Table 3).

**Table 3.** Thematic Analysis Summary: Facilitators of Psychological Safety

Themes	Definition	Subthemes and representative quotes
Work involved in promoting psychological safety	Promotion of psychological safety requires intentionality and deliberate actions from all team members, including trainees themselves, chief residents, and faculty	Deliberate actions: “So, I think it goes beyond what is specifically being taught but more of a continued conversation about education is important, trainees are important, feedback is important. Developing a safe space in the operating room is important. I think the kind of more you talk about it, the more you do it, the more you walk and talk it, the more buy-in faculty will have.” (Participant 107) Familiarity and relationships: “So, I think that a lot of it is social bonds, that if you just get to know each other, just a little bit, that can really go a long way towards building the team and, and the psychological safety.” (Participant 102) Role of chief residents: “I had chiefs that took the time to teach me, gave me the confidence to make a little bit of mistakes and learn from my mistakes. There’s not that sense of competition here like the idea like someone’s win means your loss. And I think fostering a sense of ‘there’s enough for everyone’ helps people feel like, oh, just because someone got the sweet case or oh because someone is like bonding with this attending doesn’t necessarily mean that’s going to take away from my education.” (Participant 100)
Trust	Instances where trust was initially “on the line” (ie, at serious risk of being lost) but ultimately reinforced formed the basis for psychological safety	“I guess the thing that comes to my mind is, to me psychological safety means trust, like, do I trust you or not? And trust is something that is not a right or wrong answer. It’s a connection that is built… the more opportunities you give me to do something, and you continue to interact with me whether or not I do it perfectly right or not, like, that builds psychological safety. So, there’s a certain amount of like, I just have to have interactions with you. But I also have to have interactions that are risky.” (Participant 113) “And I remember that sense of her like understanding I made a mistake but not making me feel worse about it let me know that I could trust her. Even when things were going left or that she could trust my clinical judgment, or she didn’t believe I was a failure…” (Participant 100)
Normalizing vulnerability in the clinical learning environment	Other trainees and faculty openly admitting faults and sharing mistakes normalized vulnerability and asking for help	Sharing shortcomings: “And I’ve been very vocal about everything I’ve gone through. And I tell my interns, anyone, you know, look at what I’m going through, and I can still do these things. Don’t let people tell you otherwise because I remember looking at the chiefs when I was a PGY-1 and 2, and being like, ‘Oh my God, they’re perfect.’ I just didn’t feel a lot of vulnerability. So then when I started to really struggle, I’m like, well, am I alone in this? Or is this normal? There are so many parts of residency where you feel so isolated and alone and you can’t talk about it. And then I think that little by little [it] just digs and chips away at your ability to have psychological safety.”(Participant 115) Openness: “You know, I think it’s inherent to our profession that our mistakes are going to be higher stakes. I think not normalizing the harm but normalizing the fact that mistakes do happen is one of the things I specifically do on my team. So that people rather than trying to hide mistakes… it’s my thought that if I normalize it, it’s my things that I normalize, people are less likely to hide mistakes from me.” (Participant 105) Giving grace: “I think when people know where their leader is at, I think it changes their tone. Let’s say you go rounding and, you know, you forget to look at a drain. If I was an intern on a service where the chief is supposed to be this perfect human who never eats or sleeps, she’s supposed to be this perfect human, I’d be like ‘Oh wow, that chief forgot about that drain. How could they do that?’ And then, you know, if you have a little more context you can be thinking maybe, give people a bit more grace that you forget to do something… some of the mundane parts of the job that are still important, but they might get missed when you’re in a hurry or if people aren’t operating at their best.” (Participant 105)

Abbreviation: PGY, postgraduate year.

#### The Work Involved in Promoting Psychological Safety

Trainees overwhelmingly described the intentionality necessary to promote psychological safety. Three subthemes were identified: (1) deliberate actions, (2) familiarity and relationships, and (3) the role of chief residents. Trainees emphasized that fostering psychological safety required deliberate effort by faculty and residents, particularly in making the “implicit explicit.” For instance, psychological safety was actively promoted when faculty deliberately invited residents to speak up or when faculty asked and responded to questions in a nonjudgmental manner. Although this openness was often assumed by faculty, trainees noted that those who repeatedly encouraged trainees to speak up during face-to-face interactions helped explicitly promote psychological safety. Conversely, trainees also expressed personal accountability in facilitating psychological safety for themselves. Trainees described taking ownership of their education through increased receptivity to feedback, fostering safe spaces for junior residents, preparing adequately for cases, and maintaining positive relationships with operating room staff.

Familiarity and establishing relationships with faculty and coresidents formed the foundation of psychological safety. The positive associations of familiarity were evidenced by trainees’ increased willingness to speak up, reduced fear in asking questions, and greater likelihood of asking for help. Camaraderie among residents contributed to an educationally safe environment where there was a lack of competition. As 1 trainee stated, “There’s not that sense of competition here, like the idea that someone’s win means your loss… There’s enough for everyone.” Chief residents within each program also played a pivotal role in promoting psychological safety. The chief residents set the tone by actively inviting questions, responding to mistakes without blame, and fostering an open learning environment. These actions collectively facilitated psychological safety for junior trainees, even in instances when faculty themselves did not prioritize it. Paying it forward was a large motivating factor for senior residents to establish psychological safety; as 1 trainee emphasized, “be the resident you wish you had or be the attending you wish you had.”

#### Trust

The presence of trust and entrustment of responsibilities among coresidents and the relationships between residents and faculty were essential in enabling psychological safety. Interactions involving risk taking where trust was “on the line” (ie, at serious risk of being lost) but ultimately reinforced built the foundation for psychological safety. As 1 trainee stated, “The more opportunities [the faculty] give me to do something, and [they] continue to interact with me, whether or not I do it perfectly… that builds psychological safety.” This persistence of trust after mistakes, interpersonal conflicts, and delivery of constructive feedback strengthened relationships and ultimately increased psychological safety among trainees.

#### Normalizing Vulnerability in the Clinical Learning Environment

Expressions of fallibility were essential to building a psychologically safe environment. Three subthemes were identified: (1) sharing shortcomings, (2) openness, and (3) giving grace. Trainees, particularly those at a junior level, emphasized the importance of witnessing chief residents and faculty share shortcomings and openly discuss failures. Maintaining openness, in terms of team communication and providing physical spaces to facilitate sharing of vulnerabilities, further promoted psychological safety. In scenarios where mistakes were made or trainees asked for help, nonjudgmental validation of these efforts and offering grace were imperative.

## Discussion

In this multi-institutional qualitative study, we identified key individual, interpersonal, and organizational factors within the learning environment associated with psychological safety for surgical trainees. Our results highlight the complex nature of psychological safety as identified barriers and facilitators spanned broadly across the learning environment. These findings may help inform the future development of interventions aimed at improving psychological safety for surgical trainees.

To our knowledge, this is the first study to examine surgical trainee perceptions of barriers and facilitators of psychological safety on a multi-institutional level. Although psychological safety has been explored in surgical teams and related health care settings,^5,17,22^ few studies have evaluated its role specifically for trainees. The inherent training structure and apprenticeship model of surgical residency often create a learning environment not conducive to speaking up and voicing concerns. In our study, key barriers to psychological safety among trainees centered around perceived learner mistreatment, judgment from educators, and fear of potential judgment for knowledge or skill deficits. Given the associations of mistreatment with trainee burnout and reduced well-being,^23,24^ it is not surprising that actions such as bullying, microaggressions, and incivility contribute to a psychologically unsafe environment. For instance, the work by Dyurich and colleagues^25^ defined mistreatment in surgical training as “personal, public, and/or persistent” actions that resulted in residents feeling “demeaned, dismissed, and/or disrespected.” The authors noted that the presence of psychological safety was a key mitigator of mistreatment. In addition to mistreatment, our findings also highlight the perceived importance of reputation in psychological safety as the fear of tarnishing or damaging one’s reputation effectively hindered speaking up. Instead of asking questions and identifying knowledge deficits, learners felt the need to constantly self-monitor for mistakes in settings that lacked psychological safety. Consequently, the ubiquitous sense of risk in the learning environment prevented trainees from fully immersing themselves in the learning experience.

In contrast, we identified several facilitators of psychological safety. A novel finding of this study is the importance of risk taking in the trainee-faculty relationship to promote psychological safety. Interactions that initially required trainees to step outside of their comfort zone and were subsequently met with acceptance and positive reinforcement from educators led to increased trust in the learner-educator relationship. Consequently, this positive feedback cycle formed the building blocks of psychological safety. Additional factors associated with psychological safety included intentionality, entrustment, and open displays of vulnerability. Potential targeted interventions to incorporate these findings in clinical practice include educational workshops, increasing familiarity between trainees and faculty, facilitated focus group discussions, and prioritizing psychological safety at the program and leadership levels.^26^ There are currently few studies to date that have examined interventions to increase psychological safety exclusively among surgical trainees. Among studies that have implemented interventions such as simulations and workshops within surgical teams, there is a lack of objective outcome measures to accurately evaluate efficacy. In particular, the SECOND (Surgical Education Culture Optimization Through Targeted Interventions Based on National Comparative Data) and THIRD (Trial to Harness Inclusion and Foster Resilient Departments of Surgery) trials have implemented intervention toolkits to support resident well-being.^27^ Moving forward, the thematic analysis presented here may be used to inform the development of a similar toolkit of targeted interventions to optimize psychological safety for surgical trainees.

A key distinction highlighted by our findings is the difference between psychological safety and comfort. There often exists a misperceived notion that a psychologically safe learning environment is one that caters to learners and their needs. However, as noted by trainees in our study, a learning environment that fails to clinically challenge learners is ultimately detrimental to trainee growth and development. For instance, despite fearing judgment for answering questions wrong, trainees expressed a desire for faculty to ask more questions, as this presented an opportunity to build trust in the trainee-educator relationship. However, prioritization of comfort over challenging interactions in the learning environment often led to loss of learning opportunities, as knowledge and skill deficits persisted in the absence of constructive feedback. Furthermore, although this study focused on trainee experiences with psychological safety, further exploration of faculty perspectives in the clinical learning environment is warranted. Though trainees perceived certain faculty actions as mistreatment, it is unlikely that these actions were ill intentioned. As prior studies have shown that trainee and faculty perceptions may differ significantly in the clinical learning environment,^28,29^ incorporation of faculty perspectives will be essential for alignment of expectations in the trainee-faculty relationship.

Our findings add to the currently limited literature on psychological safety among surgical trainees.^16,17,22,30,31,32^ Ojute and colleagues^16^ conducted focus groups to examine enhancers and inhibitors of support among 28 surgical residents. Allyship of mentors and nonpunitive analysis of mistakes were major enhancers, while system and cultural factors were key inhibitors of support within the workplace. Pychological safety was identified as a prominent theme within enhancers of support. Our results align with these findings and add specificity to behaviors within the clinical learning environment in association with psychological safety. The importance of interpersonal relationships and familiarity were also noted to be key factors associated with psychological safety in a survey study across 7 emergency medicine residency programs.^17^ Furthermore, establishing a psychologically safe learning environment begins early in medical training.^10,33,34^ As McClintock and colleagues^34^ have noted, educator behaviors such as dismissal of questions, lack of educator interest, and withholding autonomy served as detractors of psychological safety. These findings show that prioritization and promotion of psychological safety must begin early, as its effect on trainees and their learning behaviors are long lasting and carry over into residency.

### Limitations

This study should be interpreted in the setting of its limitations. First, a small proportion of trainees in our study were underrepresented in medicine, which may limit generalizability of our findings across racial and ethnic minority groups. However, in keeping with the methodological rigor for qualitative research, the transferability of findings is meaningful. As these racially and ethnically marginalized groups are often at the highest risk of mistreatment, future studies are needed to explore their unique training experiences in association with psychological safety. Second, there may be selection bias, as trainees with extreme experiences, whether positive or negative, could have been more inclined to participate in our study. Third, our study has the potential for interviewer bias because 2 authors (J.H.C. and A.A.P.) are current or former general surgery trainees and 2 authors (J.E. and G.S.) are qualitative researchers in surgical education. To mitigate bias, reflexivity was practiced throughout the study design and analysis using written reflections, collaborative team discussions, and constant comparative analysis. Fourth, as the learner-educator relationship is a 2-way street, incorporating perspectives of faculty educators in psychological safety will be essential.

## Conclusions

This qualitative study identified 7 key individual, interpersonal, and organizational factors associated with psychological safety in the clinical learning environment for surgical trainees. Our findings serve as important building blocks for future interventions aimed at increasing psychological safety among surgical trainees.^26^ Although the focus remains on optimizing the learning environment for trainees, increased psychological safety in the workplace may also improve team dynamics, encourage communication, and ultimately prevent surgical errors and improve patient outcomes.^22,35,36^ As such, we as a surgery community have a responsibility to prioritize psychological safety, not only to support future generations of surgical trainees, but also for the benefit of our patients.

## Notes

Supplement 1. **eAppendix.** Semi-Structured Interview Guide

Supplement 2.
Data Sharing Statement
