# Supplementary material for: Barriers and Facilitators to Surgical Trainee Psychological Safety
Source: JAMA Netw Open. 2025 Sep 29;8(9):e2534462. doi: 10.1001/jamanetworkopen.2025.34462 (PMC12481224; doi:10.1001/jamanetworkopen.2025.34462)
Supplement: Supplement 1. — eAppendix. Semi-Structured Interview Guide [file jamanetwopen-e2534462-s001.pdf]

Supplementary Online Content

Chen JH, Pradarelli AA, Evans J, et al. Barriers and facilitators to surgical trainee psychological safety. *JAMA Netw Open.* 2025;8(9):e2534462. doi:10.1001/jamanetworkopen.2025.34462

eAppendix. Semi-Structured Interview Guide

This supplementary material has been provided by the authors to give readers additional information about their work.

© 2025 Chen JH et al. *JAMA Network Open.*

eAppendix. Semi-Structured Interview Guide

Introduction:

Thank you for agreeing to answer some questions about your experience in the learning environment at your institution. As a reminder, this interview is confidential. There are no right or wrong answers. You can choose to skip any questions at any time. Our goal in this interview is to hear about your experiences and perspectives. We are not trying to prove something or further an agenda. The interview will be recorded for transcription purposes. Once the transcriptions have been de-identified, the recordings will be destroyed. Do I have your permission to record? What questions do you have before we begin?

1. If you met someone who had never been to your program before, what would you tell them about the learning environment?
    a. Follow-up Prompt: You mentioned ___________________. Can you tell me more about that?

2. **Psychological safety**
    People do their best when they believe they "will not be punished or humiliated for speaking up with ideas, questions, concerns, or mistakes, and that the team is safe for interpersonal risk taking." This is referred to as psychological safety.

    a. What does it look like when there has been psychological safety in your learning environment?
        i. Can you offer an example that illustrates this?
        ii. What kinds of things contributed to this safe educational space?

    b. What does it look like when things have not felt psychologically safe in your learning environment?
        i. Tell me about an experience that captured this.
        ii. What gets in the way of promoting psychological safety?

3. **Personal performance worry**
    a. As trainees, we often worry about our performance at work. What does performance worry look like for you?
        i. How does this affect how you interact with attendings? Other residents?
        ii. How would your training experience change if you did not have performance worry?

4. **Feedback**
    Feedback is an important part of the learning process. As trainees, often we receive helpful feedback that advances our skills. Other times, we receive unhelpful or even unfair feedback.

    a. What are your thoughts about that?
    b. What experience with feedback comes to mind?
    c. How did that experience affect your interactions in the learning environment? How did it impact your willingness to try new things or take interpersonal risks?

5. **Autonomy and independence**
    The degree of autonomy and independence you receive as a trainee can be very impactful on your experience.

© 2025 Chen JH et al. *JAMA Network Open.*

a. At your stage in training, what does appropriate clinical autonomy look like for you?
b. Give us an example of a time when you were given appropriate autonomy. How did this impact your learning?
c. What about a time when you were given insufficient autonomy? How did this impact your learning?

6. **Disrespect and retaliation**
Some trainees we've talked to have said they experience disrespect and worry about retaliation in the learning environment, and this affects the feedback they provide to attendings and the program. Other trainees say they don't worry about the disrespect or retaliation in giving the feedback they want to give.
   a. What do you think about those ideas?
   b. Give us an example of a time you experienced disrespect. How did this impact your learning?
   c. How has fear of retaliation impacted your experience in training?

7. **Professional well-being**
Definition for our purposes: Professional well-being is the experience of positive perceptions and the presence of constructive conditions at work and beyond that enables workers to thrive and achieve their full potential. Considered a function of job satisfaction, finding meaning in work, feeling engaged and fulfilled with work, having a high-quality working life and professional fulfillment.[1,2]
   a. What does professional well-being look like for you on a good day?
   b. How would you compare your professional well-being now compared to when you first started residency?
   c. Sometimes our professional well-being can be compromised. What does that look like for you?
   d. How do you balance team vs individual well-being at work?

8. **Professional fulfillment**
Definition for our purposes: the degree of intrinsic positive reward we derive from our work, including happiness, meaningfulness, contribution, self-worth, satisfaction, and feeling in control when dealing with difficult problems at work.[3]
People work best when they find their work personally fulfilling and when they feel their work makes a difference.
   a. What about your work gives you the most meaning?
   b. When do you feel most valued for the work you do?
   c. When have you felt most connected to others (e.g., patients, co-workers)?

9. **Work exhaustion**
We hear about medicine being a caring profession – where the job is to care for others. Which then raises the point about who cares for the caregiver.
   a. What comes to mind when you hear that?
   b. Is there something contributing to your work exhaustion that you would really like to change?
   c. Tell me about how you have managed work exhaustion.

10. **Interpersonal disengagement**
Some trainees have described moments of exhaustion when they found it difficult to empathize with colleagues or even patients.

© 2025 Chen JH et al. *JAMA Network Open.*

a. Tell me your thoughts about this.
    i. (If they have felt this way) What experience comes to mind?
    ii. How did that affect you?
    iii. How did it affect your interactions in the learning environment?

Closing Question

11. Our hope is to use these findings to develop strategies that improve psychological safety for faculty and trainees. Based on your experience, what 1-2 suggestions do you have for improving psychological safety at your program?

© 2025 Chen JH et al. *JAMA Network Open.*
