# Supplementary material for: Barriers and Facilitators to Surgical Trainee Psychological Safety
Source: JAMA Netw Open. 2025 Sep 29;8(9):e2534462. doi: 10.1001/jamanetworkopen.2025.34462 (PMC12481224; doi:10.1001/jamanetworkopen.2025.34462)
Supplement: Supplement 2. — Data Sharing Statement [file jamanetwopen-e2534462-s002.pdf]

Data Sharing Statement

Chen. Barriers and Facilitators to Surgical Trainee Psychological Safety. *JAMA Netw Open*. Published September 29, 2025. doi:10.1001/jamanetworkopen.2025.34462

Data
Data available: No

Additional Information
Explanation for why data not available: Participants could potentially be identifiable.
